# $\textsc {McDag}$: indexing maximal common subsequences for *k* strings

**DOI:** 10.1186/s13015-025-00271-z

**Published:** 2025-04-19

**Authors:** Giovanni Buzzega, Alessio Conte, Roberto Grossi, Giulia Punzi

**Affiliations:** Dipartimento di Informatica, Università di Pisa, Largo Pontecorvo 3, 56127 Pisa, Italy

**Keywords:** Common subsequences, Inclusion maximality, Directed acyclic graph, Index data structure

## Abstract

Analyzing and comparing sequences of symbols is among the most fundamental problems in computer science, possibly even more so in bioinformatics. Maximal Common Subsequences (MCSs), i.e., inclusion-maximal sequences of non-contiguous symbols common to two or more strings, have only recently received attention in this area, despite being a basic notion and a natural generalization of more common tools like Longest Common Substrings/Subsequences. In this paper we simplify and engineer recent advancements in MCSs into a practical tool called $\textsc {McDag}$, the first publicly available tool that can index MCSs of real genomic data, and show that its definition can be generalized to multiple strings. We demonstrate that our tool can index pairs of sequences exceeding 10,000 base pairs within minutes, utilizing only 4-7% more than the minimum required nodes. For three or more sequences, we observe experimentally that the minimum index may exhibit a significant increase in the number of nodes.

## Introduction

Strings are fundamental in computer science, and their analysis, indexing, and processing are among the oldest and best-studied problems. A central aspect of these problems is searching for relevant patterns in strings, which vary based on the application. We focus here on patterns that are *common* between two or more strings.

In some real-world domains, a common substring may be too strict of a requirement: to make an example, a sequence of bases in genetic data may represent an important gene, but different specimens may have undergone different micro-variations in their genetic code that very slightly altered the gene, and even the act of sequencing introduces noise in the data so that an exact match is not guaranteed even when comparing samples from the same specimen. In these domains, it is relevant to consider the *common subsequence*: an ordered sequence of characters that occurs in all given strings, but not necessarily contiguously, i.e., the characters of the sequence may be interleaved with others.

**Fig. 1:**
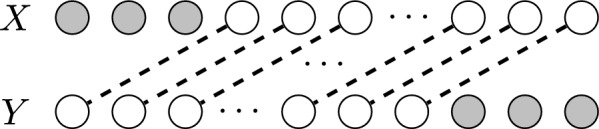
In the example considering two genetic sequences, the LCS only shows the longer white “promoter sequence”, a common occurrence in genomic sequences. The critical (but shorter) shaded common part cannot be extended to a common subsequence of the same length and is thus not shown by LCS-based analysis

As the number of common subsequences between even just two strings can be exponentially high, a common idea is looking at only the one of maximum length, the *longest common subsequence* (LCS hereafter). LCSs are used to see how well two or more sequences align, or how similar they are [1]. While LCS-based approaches can be effective, they have significant limitations: firstly, efficiency is limited as finding a single LCS among an arbitrary number of strings is NP-complete [2], and still takes quadratic time with just two strings (see the conditional lower bounds in [3, 4]). Also, Fig. 1 shows an example case where a critical but relatively short sequence cannot be extended to a common subsequence as long as an LCS, thus any analysis based on LCSs would completely disregard this information. Of course, the problem persists when considering more than two strings.

On the other extreme, it is possible to consider *all* common subsequences with a Common Subsequence Automaton [5]. However, these will include many solutions (possibly most) that are included within other longer solutions, and thus pollute the set with redundant information. For these reasons, we focus on a generalization of LCSs called *Maximal Common Subsequences* (MCS hereafter), which provides an interesting middle point between LCS and all subsequences: an MCS *S* between two (or more) strings is a sequence of characters that occurs as a subsequence in each of the strings and that is *(inclusion) maximal*, that is, *S* cannot be extended with any character in any position and still be a common subsequence. For example, in Fig. 1, the critical shorter common subsequence of two strings *X* and *Y* may be included in the set of MCSs, and could improve the alignment of some critical common parts.

Once established our interest in MCSs for string analysis, the natural question is: which tools should be employed? In fact, very little was possible until recently. We now have an algorithm to enumerate all MCSs efficiently [6] but they can be exponential in number (in fact, this is true for LCSs too [7]) and it would be inconvenient to both enumerate and store all of them. A useful tool to facilitate analysis using MCSs would be a sufficiently small index that allows us to retrieve and query MCSs efficiently.

First steps in this direction have been taken: [8] proves the existence of a DAG (Directed Acyclic Graph) of polynomial size that is able to represent all MCSs for two strings, count them, and reconstruct them as needed. Similar structures have also been shown in [9]. In this paper, we consider the general problem of representing all MCSs for $k \ge 2$ strings and present an extended version of our preliminary results in [10].

### Contribution

Based on these results, the contribution of this paper is to build an indexing tool, which we call $\textsc {McDag}$, that is simultaneously simpler in concept and faster to construct than existing approaches. Moreover, while $\textsc {McDag}$ was designed in the preliminary version of this paper [10] to index MCSs between two strings, in this paper we further generalize it to be able to index MCSs between *k* strings, $MCS(\mathcal {Z})$, where $\mathcal {Z}$ is a collection of $|\mathcal {Z}| = k\ge 2$ strings. This is the first tool able to perform such task, to the best of our knowledge.

**Fig. 2:**
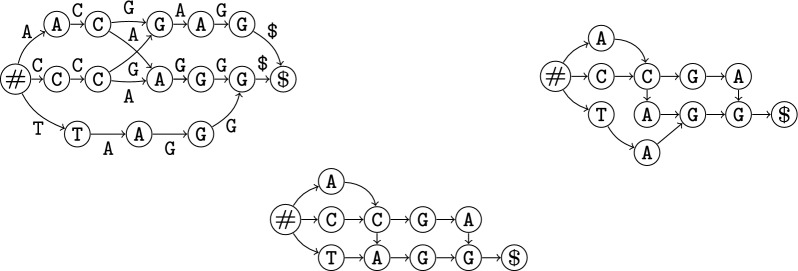
$\textsc {M-Dag}$ (on the left, taken from [8]), $\textsc {CSA-maximal}$ (on the right, derived from [5] and our filtering methods), and $\textsc {McDag}$ (at the bottom, this paper), for input strings $\mathcal {Z}_1=$ TCACAGAGA and $\mathcal {Z}_2=$ ACCCGTAGG. Here, $MCS(\mathcal {Z}) =$ ACGAG, ACAGG, CCGAG, CCAGG, TAGG

For input strings $\mathcal {Z} = \{\mathcal {Z}_1, \mathcal {Z}_2\}$, Fig. 2 shows our proposed $\textsc {McDag}$ at the bottom, along with two other DAGs obtained from the literature [5, 8].^1^ Apart from the different number of nodes and edges, all share the same conceptual structure:

- There exists a single source *s*, labeled with marker #, and a single sink *t*, labeled with marker $. All other nodes are labeled with characters from the alphabet $$\Sigma $$ of the strings in $$\mathcal {Z}$$.
- Each *st*-path is associated with a unique string in $$MCS(\mathcal {Z})$$ spelled out in its traversed nodes; vice versa, each string in $$MCS(\mathcal {Z})$$ has a unique *st*-path associated.
- The out-neighbors of each node are labeled with distinct characters from $$\Sigma $$, so the out-degree is at most $$|\Sigma |$$ (ignoring # and $ labeling *s* and *t* respectively).

As a result, each prefix of an MCS has a unique path from *s*. For example, ACA is found in $\textsc {McDag}$ following #, A, C, and A in this order, each time with a unique branching choice on the current node. The $\textsc {McDag}$ for $k=2$ has less than $|\mathcal {Z}_1| \times |\mathcal {Z}_2|$ nodes in our experimental study of Sect. 4, only 4-7% more than the minimum required nodes. It takes quadratic time in practice, which allows us to index sequences exceeding 10,000 base pairs within minutes. Note that, in general, no DAG storing $MCS(\{\mathcal {Z}_1,\mathcal {Z}_2\})$ can take sub-quadratic time in the worst case unless SETH or OVH fail, as an LCS is an MCS of maximum length, and the problem of finding the LCS length has a quadratic conditional lower bound [3, 4].

In general, for $k = |\mathcal {Z}| \ge 2$, we can extend our argument and conjecture that $\textsc {McDag}$ has $O(n^k)$ nodes, assuming without loss of generality that all strings in $\mathcal {Z}$ have the same length *n*. A smaller number of nodes is unlikely, as $O(n^k)$ time is required to find an LCS of the string set $\mathcal {Z}$ under a conditional lower bound from fine-grained complexity. However, our experiments in Sect. 4 indicate that the number of nodes in $\textsc {McDag}$ for $k > 2$ could be exponentially large in *n*, contrary to our conjecture, which is an interesting observation.

In any case, the benefit of the above conceptual structure is that it fits several efficient algorithms on the state of the art for querying deterministic acyclic automata. For instance, listing all the strings in $MCS(\mathcal {Z})$, reporting only those of (up to) a given length, or matching a simple regular expression, and counting the number of the above strings (e.g. see [8]). We argue that $\textsc {McDag}$ is a significant first step in this type of analysis. It is—to the best of our knowledge—the first publicly available tool that allows for efficiently indexing and analyzing MCSs and can process sequences of over 10000 symbols in just a few minutes (https://github.com/giovanni-buzzega/McDag). While of course complex genes such as human ones are orders of magnitude longer and require further development of the tool, this already allows for a deeper analysis of simpler genomic data or selected segments.

### Related work

The concept of MCSs first appeared in a general form in the data mining community [11]. In this context, the authors considered ordered sequences of sets of items rather than strings. A subsequence is obtained from a sequence by deleting any number of items from any set at any position. The focus was on finding frequent subsequences, which are subsequences that appear in more than a user-defined number of sequences in the database. One of the problems proposed was to find inclusion-maximal frequent subsequences, which are not subsequences of any other frequent subsequences. Our problem can be seen as a special case of this framework by considering $k \ge 2$ sequences of singletons and setting the frequency threshold to *k*.

The MCS problem was later formalized in [12], along with several variations of the common subsequences, for which they studied the computational complexity and dynamic programming solutions in some cases. Further solutions to this problem have been proposed in various studies. Sakai provided the first (almost) linear-time algorithm to extract one MCS between two strings [13]. Bulteau et al. [14] used MCSs as a tool for a new parameterized LCS algorithm. Hirota and Sakai explored MCSs for multiple strings [15]. Conte et al. [8] and Hirota and Sakai [9] independently proposed DAGs for enumerating MCSs of two strings. Conte et al. [8] published the first polynomial-size DAG in the literature, where each node represents at least one prefix of some MCS.

We give some detail of the latter: if there is an edge from node *u* to node *v*, all prefixes of *u* are prefixes of some MCS and when extended with the character associated with that edge they do not lose this property. This allows for the direct construction of an MCS index, but maintaining it can be costly [6], as finding the right character to extend a prefix may require expensive computation. For instance, finding a character that extends an MCS prefix to a common subsequence prefix is simple, but it may yield prefixes that do not lead to any MCS. The current approach for finding suitable extensions associates a distinct quadruple of integers to each node, causing the automaton size to be $n^3$ or more in terms of nodes.

Subsequence-related problems have been previously addressed using automata. Baeza-Yates [16] introduced the Directed Acyclic Subsequence Graph (DASG), which accepts all subsequences of a given string and can be generalized to accept subsequences of any string in a set. A subsequent result was the common subsequence automaton (CSA) [5, 17, 18]: it accepts common subsequences of a set of strings, including non-maximal ones, and it is similar in concept to the common subsequence tree of [19]. The CSA can also be used to find an LCS between two strings [20]. Moreover, automaton-inspired tools such as binary decision diagrams like ZDD [21] and SeqBDD [22] can be used to compactly represent the set $MCS(\{\mathcal {Z}_1, \mathcal {Z}_2\})$, but construction is non-trivial: one potentially needs to first generate all MCSs and this can take exponential time and space.

As for LCSs, some algorithms for their computation can be seen as dynamic programming on some DAG [23, 24]. Furthermore, a DAG representation of LCSs was also recently used in [25] for the problem of finding diverse LCSs.

### Paper organization

In the rest of this section, we first introduce some preliminary notions employed in the paper. In order to describe our index $\textsc {McDag}$ in a clear way, we need to go by steps.

We will start in Sect. 2 by presenting a procedure, $\textsc {McConstruct}$, which constructs a deterministic MCS index, given in input an *approximate* one, that is, an index that contains all MCSs, but also contains non-maximal common subsequences. Indeed, $\textsc {McConstruct}$ can generate different MCS indices, with $\textsc {McDag}$ among them, depending on the input. Section 2.1 will present one possible simple input, to generate a first MCS index $\textsc {CSA-maximal}$. The $\textsc {McConstruct}$ algorithm is of independent interest, and of non-trivial correctness proof, which is given in Sect. 2.2.

In Sect. 3 we will describe the optimized input index $\textsc {CSA-filtered}$ for the $\textsc {McConstruct}$ procedure, which will finally give rise to $\textsc {McDag}$ as output. Once again, the correctness proofs are separately given in Sect. 3.2.

We conclude by presenting our experimental analysis in Sect. 4, showcasing the practical advantages of the optimized $\textsc {McDag}$ with respect to both the initial index $\textsc {CSA-maximal}$, and the previously existing index from [8]. We discuss index sizes and construction time for the case of two strings, and we analyze the size of the minimal MCS index in the general case of $k > 2$ strings.

### Preliminaries

We consider a string $X=X[1]\ldots X[|X|]$ as a sequence of characters from an alphabet $\Sigma$, where $X[j]\in \Sigma$ denotes the character at position *j* in *X* and |*X*| denotes the total number of characters in *X*. We use special characters $\{\#,\$\}$ as markers delimiting input strings.

A string *W* is a subsequence of *X* if there exist indices $1 \le j_1< \dots < j_{|W|} \le |X|$ such that $X[j_h] = W[h]$ for $1 \le h \le |W|$. Let us consider a collection of *k* strings $\mathcal {Z}=(\mathcal {Z}_i)_{i=1}^k$. *W* is a common subsequence of strings $\mathcal {Z}$ if *W* is a subsequence of $\mathcal {Z}_i$ for all *i*. We say that a *k*-ple $m = (m_i)_{i=1}^k$ is a *match* if there is a character $c\in \Sigma$ such that $\mathcal {Z}_i[m_i] = c$ for all *i*; for short we identify $c = \mathcal {Z}[m]$. We can define a partial order relation between matches as follows: we say that $m < m'$ if and only if for each *i* we have that $m_i < m'_i$; analogously, we say that $m \le m'$ if and only if for each *i* we have that $m_i \le m'_i$. Clearly, for each common subsequence *W* of $\mathcal {Z}$ there must exist at least one sequence of matches $m^1,\dots ,m^{|W|}$ such that $W = \mathcal {Z}[m^1]\dots \mathcal {Z}[m^{|W|}]$ and $m^1<\dots < m^{|W|}$; we refer to such sequence of matches as a *matching* in $\mathcal {Z}$, with corresponding string *W*.

In our example of Fig. 2, $$W = $$ CGA is a common subsequence of $$\mathcal {Z}_1=$$ TCACAGAGA and $$\mathcal {Z}_2=$$ ACCCGTAGG, and one of its matchings is underlined in $$\mathcal {Z}_1$$ and $$\mathcal {Z}_2$$ as (2, 3), (6, 5), (9, 7).

We say that *W* is a *longest common subsequence* (LCS), or belongs to $LCS(\mathcal {Z})$, if there is no common subsequence that is strictly longer than *W*. Finally, *W* is a *maximal common subsequence* (MCS) of $\mathcal {Z}$ if there is no string *X* that satisfies both conditions: *(i)* *X* is a common subsequence of $\mathcal {Z}$, and *(ii)* *W* is a proper subsequence of *X*. The set of all strings that are maximal common subsequences is denoted by $MCS(\mathcal {Z})$. Note that $LCS(\mathcal {Z}) \subseteq MCS(\mathcal {Z})$, as an LCS is an MCS of maximum length.

We next introduce some graph notions. A *directed graph* $G=(V,E)$, where *V* is the set of nodes and $E\subseteq V\times V$ is the set of edges, is a graph so that each edge (*u*, *v*) has a direction from *u* to *v*. Specifically, two edges (*u*, *v*) and (*w*, *z*) are adjacent if $v=w$. A path in *G* is a sequence of distinct edges, each adjacent to the next. If the path starts at node *s* and ends at node *t*, it is called an *st*-path; it is a cycle when $s=t$. A *DAG* $G = (V,E)$ is a directed acyclic graph. Given a node *u*, the set $N^+(u)$ indicates the out-neighbor nodes *v* such that $(u, v) \in E$, and the set $N^-(u)$ indicates the in-neighbor nodes *v* such that $(v,u) \in E$. The out-degree of *u* is $d^+(u) = |N^+(u)|$, and its in-degree is $d^-(u) = |N^-(u)|$; *u* is a source if $d^-(u) = 0$, and a sink if $d^+(u) = 0$. We consider a *labeled* DAG $G=(V,E,\ell )$, where each node *u* is associated with a character $\ell (u) \in \Sigma \cup \{\#,\$\}$.

### Definition of index for MCS

#### Definition 1

(Index for MCS) Given a collection of strings $\mathcal {Z}=(\mathcal {Z}_i)_{i=1}^k$, a labeled DAG $G=(V,E,\ell )$ is an index for $MCS(\mathcal {Z})$ if the following conditions hold:

1. Each node *u* (other than source or sink) is associated with match *m*(*u*) and has label $\ell (u) = \mathcal {Z}[m(u)]$, where $1 \le m(u)_i \le |\mathcal {Z}_i|$ for each $i \in [1\dots k]$.
2. There is a single source *s* and a single sink *t* with special values $\ell (s) = \#$ and $\ell (t) = \$$ and matches $m(s)_i = 0$ and $m(t)_i = |\mathcal {Z}_i|+1$ for each $i \in [1\dots k]$.
3. Each *st*-path $s,u_1,\dots ,u_h,t$ is associated with a unique string $W = \ell (u_1)\dots \ell (u_h) \in MCS(\mathcal {Z})$.
4. The endpoints *u* and *v* of edge $(u,v) \in E$ have associated matches such that $m(u) < m(v)$.
5. Each $W\in MCS(\mathcal {Z})$ has a corresponding *st*-path $s,u_1,\dots ,u_h,t$ such that $W = \ell (u_1)\dots \ell (u_h)$.

#### Definition 2

(Approximate index) We say a labeled DAG is an *approximate* index for $MCS(\mathcal {Z})$ when condition 3 is relaxed, so that *W* is not necessarily maximal, and so there could be *st*-paths in the DAG that store non-maximal common subsequences. All other conditions must hold.

#### Definition 3

(Determinism and co-determinism) We say that the DAG is *deterministic* if each node has out-neighbors labeled with distinct characters (and so its out-degree is at most $|\Sigma |$ and each prefix of an MCS has a unique path from *s*), and *co-deterministic* if the condition applies to the in-neighbors of each node (which has in-degree at most $|\Sigma |$). In both cases, there cannot be two distinct *st*-paths corresponding to the same string.

The DAGs in Fig. 2 are all deterministic indices for the same set $MCS(\mathcal {Z})$, and they all satisfy the above conditions. The leftmost is called $\textsc {M-Dag}$ and has been introduced in [8]. The one on the right is called $\textsc {CSA-maximal}$ and has been derived from the Common Subsequence Automaton [5] by filtering out the non-maximal common subsequences, as we shall explain in Sect. 2. The bottom one is $\textsc {McDag}$, our proposed index that further reduces the number of nodes.

#### Definition 4

(Rightmost and Leftmost indices) An (approximate) MCS index is *rightmost* if, for each edge (*u*, *v*) and for each *i*, there is no occurrence of character $\ell (u)$ in string $\mathcal {Z}_i[m(u)_i+1] \dots \mathcal {Z}_i[m(v)_i-1]$. In other words, starting from match *m*(*v*), the match *m*(*u*) is constructed using the rightmost occurrences of character $\ell (u)$. The *leftmost* property is analogously defined for character $\ell (v)$.

When any of the above deterministic indices for the set $MCS(\mathcal {Z})$ is available, a number of classical operations can be supported. For instance:

- List all the strings in $MCS(\mathcal {Z})$.
- Report only the strings in $MCS(\mathcal {Z})$ of (up to) given length.
- List the strings in $MCS(\mathcal {Z})$ containing a given string *S*, or similar regular expressions.
- Count the number of the above strings (all kinds).

We refer the reader to [8] for these operations, which can be implemented using standard algorithms from the literature on strings and automata, following the above definition of a deterministic index for MCS. As a final remark, the associated matches *m*(*u*) for the nodes *u* in the DAGs are not strictly necessary for these operations but help to quickly reconstruct a possible matching of a given MCS *W* of $\mathcal {Z}$.

## Deterministic MCS index

**Fig. 3:**
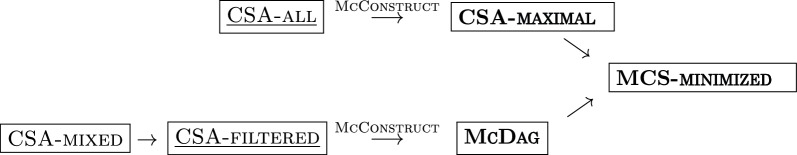
Computational paths for constructing MCS indices. Names in bold indicate deterministic MCS indices, while underlined names denote co-deterministic rightmost approximate indices. $\textsc {CSA-mixed}$ is a deterministic leftmost approximate MCS index. The top row represents our method from Sect. 2, and the bottom row shows the optimized version from Sect. 3.1. All MCS indices can ultimately produce a unique minimal deterministic MCS index, referred to as $\textsc {MCS-minimized}$

In order to define the $\textsc {McDag}$ index for input string set $\mathcal {Z}$, we need to introduce $\textsc {McConstruct}$. This procedure takes as input an approximate co-deterministic rightmost index *A* for $MCS(\mathcal {Z})$, and generates a deterministic MCS index eliminating from *A* the non-maximal sequences. We stress that the time and space complexities of the $\textsc {McConstruct}$ procedure, and of the final index, will depend on the chosen input index *A*. This is why, for ease of presentation, we will start by presenting in this section an immediate construction of an approximate co-deterministic rightmost index to be used as input for $\textsc {McConstruct}$, $\textsc {CSA-all}$. Later, in Sect. 3, we will present a more optimized version of $\textsc {CSA-all}$, $\textsc {CSA-filtered}$. By using this optimized index as input for $\textsc {McConstruct}$ we will obtain our final index $\textsc {McDag}$. We summarize all proposed indices and sketch their construction steps in Fig. 3.

**Algorithm 1:**
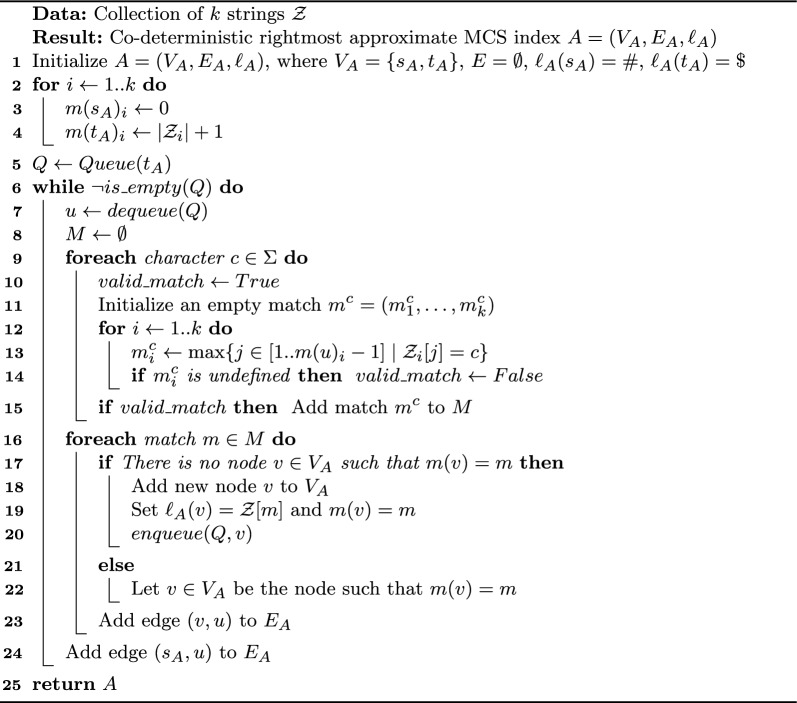
$$\textsc {CSA-all}$$ construction

### CSA-all: approximate co-deterministic rightmost index

We show how to build an index $A = (V_A, E_A,\ell _A)$ with source $s_A$, so that *A* is rightmost. We can obtain an instance of *A*, which we call $\textsc {CSA-all}$, as a vanilla version of the Common Subsequence Automaton [5] as follows (see also Algorithm 1). Since we want it to be co-deterministic, we read each string $\mathcal {Z}_i$ from right to left, and start building *A* from its sink $t_A$ backwards. Consider the generic step for a node *u*, initially $u=t_A$ with $m(t_A)_i = |\mathcal {Z}_i|+1$, for all *i*. We need to link *u* to its in-neighbors, possibly creating some of the latter ones, which are at most $|\Sigma |+1$, one per character *c* and one for the source. Hence, for *c* we find the largest match $m^c < m(u)$ such that $c = \mathcal {Z}[m^c]$: if a node *v* with $m(v) = m^c$ does not exist, we create *v* with $m(v) = m^c$ and $\ell _A(v) = c$; in any case, we add edges (*v*, *u*) and $(s_A,u)$.

The following lemma proves the correctness of the described procedure:

#### Lemma 1

The resulting $\textsc {CSA-all}$ *A*, output of Algorithm 1, is a co-deterministic rightmost approximate MCS index.

#### Proof

By construction, an edge (*u*, *v*) is added to *A* only if $m(u) < m(v)$, so *A* is acyclic by the antisymmetry of <. This also implies that all strings encoded in an *st*-path $s_A, u_1,\dots ,u_h,t_A$ are common subsequences of $\mathcal {Z}$, as there exists a matching $m(u_1),\dots ,m(u_h)$. Moreover, for all $i \in [1..k]$, $m(u)_i$ is defined as the rightmost occurrence of $\ell _A(u)$ in $\mathcal {Z}_i[1]\dots \mathcal {Z}_i[m(v)_i-1]$ before $m(v)_i$, so *A* is rightmost.

Each node *u* is visited at most once (it is added to the queue when it is added to the graph), and at most one node per character is added as in-neighbor, so *A* is co-deterministic. Node $s_A$ is the unique source, as all nodes except $s_A$ are visited and are assigned at least one in-neighbor, namely $s_A$. Node $t_A$ is the only sink, as there cannot exist any match *m* such that $m(t_A) < m$, so it cannot be added as an in-neighbor of any other node. Properties 1 and 2 are trivially granted by construction.

Now we show by contraposition that index *A* contains all common subsequences of $\mathcal {Z}$. Consider a string *W* that is not spelled by any path of *A*; let $P=w_h,\dots ,w_{|W|},t_A$ be the longest path that spells out a suffix of *W*. By co-determinism, *P* is unique, and since *A* is also rightmost, for each $j \in [h..|W|]$ and each $i \in [1..k]$, the *i*-th entry of match $m(w_j)$ defines the shortest suffix of $\mathcal {Z}_i$ that contains $W[j]\dots W[|W|]$. Since *W* is not contained in *A*, then there exists a string $\mathcal {Z}_i$ such that $W[h-1] \not \in \{\mathcal {Z}_i[1],\dots ,\mathcal {Z}_i[m(w_h)_i-1]\}$. But this means that *W* is not a subsequence of $\mathcal {Z}_i$, so it is not a common subsequence of $\mathcal {Z}$. Since all MCS are common subsequences, we have property 5 and we are done. $\square$

**Complexity. ** Apart from its source and sink, *A* has as many nodes as the matches involved in its construction. Let $n = \max _i |\mathcal {Z}_i|$; in the following, we assume wlog $n \ge |\Sigma |$. Since there cannot be two distinct nodes *u* and *v* of *A* with the same match $m(u) = m(v)$, we derive that $|V_A| \le n^k + 2$ and $|E_A| \le (|\Sigma |+1)(n^k + 1)$ as the maximum in-degree is $|\Sigma |+1$. Its construction time is $O(n^k |\Sigma | k)$, and its space is $O(n^k |\Sigma |)$.

### The McConstruct Algorithm

We now present our procedure for extracting maximal subsequences from any input approximate co-deterministic rightmost index: it is called $\textsc {McConstruct}$ and is a key component of $\textsc {McDag}$. Let $A=(V_A,E_A,\ell _A)$ with source $s_A$ be such an index; we apply Algorithm 2 to obtain a graph $G=(V,E,\ell )$ with source *s* and sink *t*, that only indexes MCS. During the construction we associate each node $u \in V$ with a set *F*(*u*) of nodes from $V_A$, all having the same label as *u*’s (initially $F(s) = \{s_A\}$ with label #). At each step we expand a node $u \ne t$ with its out-neighbors, filtering out the nodes of *A* whose matches are to the right of some match $m^c>m(u)$, as they cannot lead to an MCS: $m^c$ is a witness to defy their maximality. After that, we create new nodes in *G* for the filtered set of nodes with the same label coming from *A*, and their edges in *G*. We end up having a single sink *t*, corresponding to $\$$, only occurring at the end of both strings.

**Algorithm 2:**
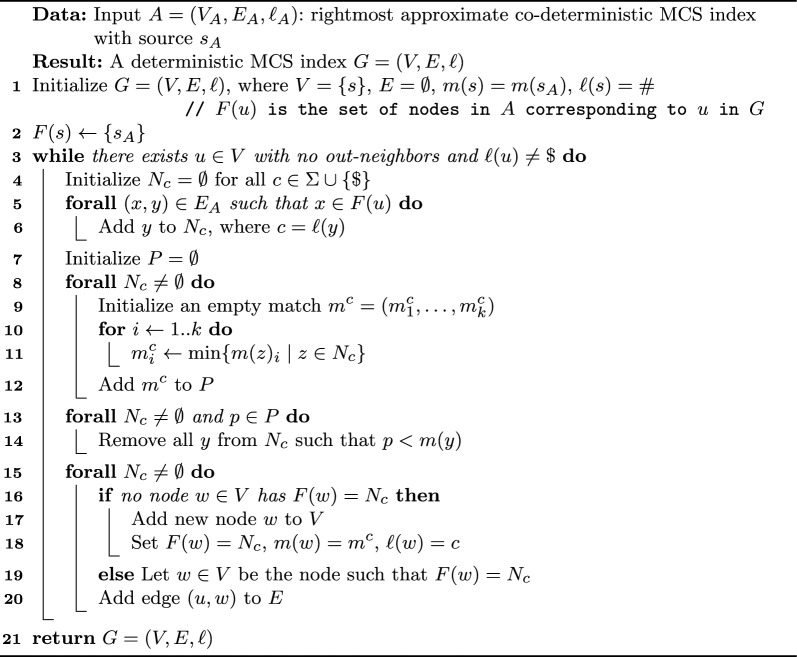
$$\textsc {McConstruct}$$

The proof of correctness of procedure $\textsc {McConstruct}$ in Algorithm 2 is non-trivial, as we have to show that we retain all and only the strings in $MCS(\mathcal {Z})$ along all the *st*-paths in *G*, and as such will be detailed in Sect. 2.3.

As for complexity, we can see from the pseudocode of Algorithm 2 that $\textsc {McConstruct}$ performs one iteration per node *v* of $\textsc {McDag}$, and, crucially, inside each iteration it performs computations for a time that is proportional to the sum of the out-degrees of each node in *F*(*v*). All such out-neighbors are then filtered and grouped by their label in the sets $N_c$, which define new nodes *w* of $\textsc {McDag}$, by setting $F(w) = N_c$. Thus, if we could reduce the number of nodes and the out-degrees of the approximate index, we would both reduce the number of nodes of the final index, as well as the amount of work done in each iteration. This observation is what motivates the improved approximate index $\textsc {CSA-filtered}$, which we present in Sect. 3.1. Moreover, it can be shown that if *A* is a co-deterministic rightmost MCS index, which can be seen as a non-deterministic finite automaton, then $\textsc {McConstruct}$ produces a deterministic finite automaton for the same language. Thus, in some cases, $\textsc {McConstruct}$ corresponds to the determinization algorithm based on the well-known powerset construction, which, in the worst case, causes an exponential blow-up in the number of nodes. Other famous algorithms such as Brzozowski’s minimization algorithm for deterministic finite automata [26] are based on such construction but are reported to perform surprisingly well in practice [27]. As Sect. 4 shows, for the case of two strings, we are in a similar situation: $\textsc {McConstruct}$ algorithm scales polynomially with respect to the length of the strings (hence also with respect to the input approximate MCS index). Interestingly, we shall observe that such behavior does not seem to hold for the general case of $k>2$ strings.

**Fig. 4:**
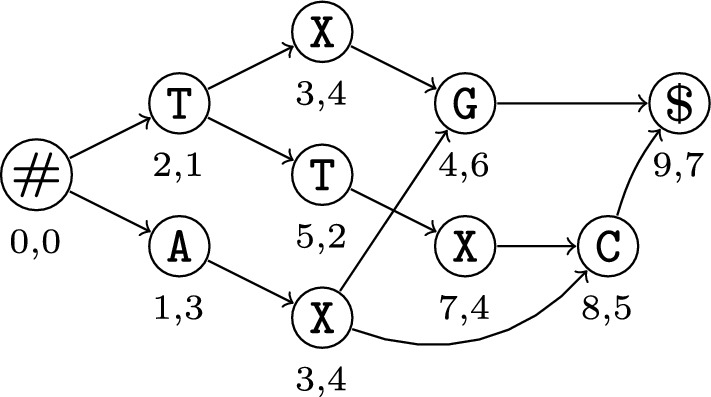
The resulting index $\textsc {McDag}$ over two input strings $\mathcal {Z}_1=$ ATXGTCXC and $\mathcal {Z}_2=$ TTAXCG. Even though it can be proven that this is the minimal deterministic MCS index, we need more nodes with label X than the possible matches of that character

In Fig. 4 we report an example of a deterministic minimal MCS index computed over two input strings $\mathcal {Z}_1=$ ATXGTCXC and $\mathcal {Z}_2=$ TTAXCG.

Remarkably, letter X only allows matches (3, 4) and (7, 4). But in order to represent all MCSs of such strings in a deterministic index, at least two distinct occurrences of match (3, 4) are needed, or else some path would represent a non-maximal common subsequence.

#### Remark 1

In a deterministic MCS index, we cannot associate each match with at most one node.

This tells us that we cannot simply bound the number of nodes with the $O(n^k)$ distinct matches in a deterministic MCS index; this is in line with the space complexity reported by Conte et al. for the case of $k=2$ strings [8].

### Correctness of McConstruct

This section is devoted to proving the correctness of algorithm $\textsc {McConstruct}$. The algorithm takes as input the output *A* of the first phase, which is an approximate co-deterministic rightmost index, and outputs a deterministic MCS index *G* as described in Sect. 2.2. First, we show a number of necessary properties that are satisfied by the output index. Then, to show that *st*-paths correspond to MCSs, we conclude the proof through a characterization of the shape of non-maximal common subsequences in similar data structures, showing that they do not occur in the index output by $\textsc {McConstruct}$.

For a set of matches $\mu = \{m^1,\dots ,m^h\}$ corresponding to the same character $c \in \Sigma$ we define their minimum as the match given by the minimum over all components: $\min (\mu ) = (\min _{1\le j\le h} m^j_i)_{i=1}^k$. For a set of nodes $\nu = \{v_1,\dots ,v_h\}$, we define their corresponding set of matches as $m(\nu ) = \{m(v_1),\dots ,m(v_h)\}$.

#### Theorem 1

During the algorithm, we retain the following properties for graph $G=(V,E,\ell )$:

1. *G* is deterministic;
2. Each node $v \in V$ is labeled with a symbol $\ell (v)$ from $\Sigma \cup \{\#,\$\}$, and is associated with a set of nodes *F*(*v*) of *A*, all labeled with $\ell (v)$. Furthermore, it is associated with match $m(v) = \min (m(F(v)))$ for character $\ell (v)$.
3. *G* is a labeled DAG with a single source *s*, having $F(s) = \{s_A\}$, $m(s) = (0,\dots ,0)$ and $\ell (s) = \#$;
4. If $$(u,v) \in E$$, then $$m(u) < m(v)$$.
5. Each path $P=s,v_1,\dots ,v_h$ in *G* is associated with unique string $str(P) = \ell (v_1),\dots ,\ell (v_h)$, which is a common subsequence of $\mathcal {Z}$ occurring at the matches of (increasing) positions $m(v_1),\dots ,m(v_h)$.

#### Proof

Conditions (1) and (2) are immediate by construction. As for (3), at the beginning node *s* is added to *G* with $m(s) = (0,\dots ,0)$ and $\ell (s) = \#$. We only add out-neighbors to existing nodes, and thus we never add new sources. Furthermore, the absence of cycles follows immediately from the same property in *A*.

Let us now recall what happens when node *u* is selected to be processed. We define the sets of possible neighbors with respect to each character for all the corresponding nodes *F*(*u*) in *A*: $N_c = \{y \in N^+(x) \ | \ x \in F(u) \text { and } \ell _A(y) = c\}$. We then filter these nodes, removing the ones with corresponding matches that come after some $\min (m(N_d))$, yielding the final set $N_c$. For each non-empty $N_c$, we consider node $w_c$ of *G* (or add it if it does not exist) such that $F(w_c) = N_c$, $m(w_c) = \min (m(N_c))$, $\ell (w_c) = c$, and add edge $(u,w_c)$ to *G*. Let us consider a newly added edge, $(u,w_c)\in E$, for some $w_c$ as defined above, and let us show that $m(u) < m(w_c)$ (property (4)). It is clear that the edge corresponds to at least one $(x,y)\in E_A$ with $x\in F(u)$ and $y\in F(w_c)$, by definition of $N_c$. Consider match $m(w_c) = \min (m(F(w_c)))$. By definition of minimum, for each position $j \in [1..k]$, there exists a node $y_j \in F(w_c)$ such that $m(y_j)_j = m(w_c)_j$. By construction, for each $y_j$ there exists one $x_j \in F(u)$ such that $(x_j,y_j) \in E_A$. Since *A* is an approximate MCS index, by property 4 we have that $m(x_j) < m(y_j)$ and in particular $m(x_j)_j < m(y_j)_j$. By definition of minimum, we have that for all *j* and $y_j$, $m(u)_j = \min (m(F(u)))_j \le m(x_j)_j < m(y_j)_j = m(w_c)_j.$ This lets us conclude that $m(u) < m(w_c)$.

Lastly, property (5) follows from the corresponding property 3 of approximate MCS index *A*: since we are never adding edges that have no corresponding ones in *A*, any path from *s* in *G* surely spells a subsequence of an *st*-path spelled by *A*, which is still a common subsequence. $\square$

Note that by construction, Algorithm 2 adds out-neighbors to all nodes *u* that have label $\ell (u) \ne \$$, hence all sinks of *G* have label $\$$. Moreover, since *A* has a unique sink, *G* must also have a unique sink, as for all nodes $|N_\$| \le 1$.

Theorem 1 together with the above observation implies that the graph output by $\textsc {McConstruct}$ satisfies conditions 1-4 of an approximate MCS index. Thus, the only things missing for *G* to be an index for $MCS(\mathcal {Z})$ are that MCSs correspond to *st*-paths and that *st*-paths correspond to MCSs. We first show that all MCSs are retained as *st*-paths of *G* (condition 5 of an MCS index):

#### Lemma 2

Each $W\in MCS(\mathcal {Z})$ has a corresponding *st*-path in the resulting graph $G=(V,E,\ell )$ at the end of the $\textsc {McConstruct}$ procedure.

#### Proof

First, recall that each MCS occurred once as an *st*-path of *A*. During the construction of *G*, we have a correspondence between edges $(u,v)\in E$ of *G*, and edges $(x,y) \in E_A$ with $x\in F(u)$ and $y\in F(v)$, which share the same respective labels. Since indeed they spell the same string, we say that an *st*-path $s_A=x_1,\dots ,x_h=t_A$ of *A* corresponds to an *st*-path $s=v_1,\dots ,v_h=t$ of *G* if $x_i \in F(v_i)$ for all *i*.

When building the neighbors of some node $u\in V$ during $\textsc {McConstruct}$, we may discard $(x,y)\in E_A$ with $x \in F(u)$, in the sense that $y \not \in F(v)$ for any $(u,v) \in E$. This happens, by construction, if and only if *y* is removed when filtering set $N_c$, that is, if and only if there exists a match $m = \min (m(N_d))$ for some $d\in \Sigma$ such that $m < m(y)$. By property (4), we further have $m(u) < m$. Therefore, we have a set of matching characters given by *m* which occur strictly between the matches given by *m*(*u*), and the ones given by *m*(*y*).

Let us now assume by contradiction that this happens for an edge (*x*, *y*) which is traversed by an MCS: let $P= s_A,x_1,\dots ,x_h,t_A$ be such that $str(P)\in MCS(\mathcal {Z})$, and let us assume that *j* is the minimum index such that there is a path $S = s,v_1,\dots ,v_j$ in *G* corresponding to prefix $s_A,x_1,\dots ,x_j$ of *P* and $x_{j+1} \not \in F(w)$ for all $(v_j,w) \in E$. By the reasoning above, there exists a match *m* such that $m(v_j)< m < m(x_{j+1})$. Let us now consider the following matching: $m(v_1),\dots ,m(v_j), m, m(x_{j+1}),\dots ,m(x_h)$. These matches are all strictly increasing (by (5) of Theorem 1 for the prefix, and by property 4 of approximate MCS index *A* for the suffix), and the consequent spelled subsequence has *str*(*P*) as a proper subsequence, a contradiction. $\square$

We now have that *G* is a deterministic approximate MCS index. To conclude the proof of correctness, the only thing left to show is that no *st*-path corresponds to a common subsequence that is not maximal. To this end, we give a characterization of the non-maximal common subsequences given by *st*-paths in approximate MCS indices. We introduce the key notion of subsequence bubbles for this purpose, and we show that we eliminate these bubbles during $\textsc {McConstruct}$. Hence non-maximal common subsequences cannot survive.

#### Definition 5

(Subsequence Bubble) Consider a DAG *D* where each node is labeled with a symbol of $\Sigma \cup \{\#,\$\}$, and let $b,e_1,e_2,e$ be four distinct nodes of *D*.

- A *closed subsequence bubble* is a pair of disjoint $\,b\,e$-paths *S*, *L* such that *str*(*S*) is a proper subsequence of *str*(*L*).
- An *open subsequence bubble* is a pair of disjoint paths, where *S* is $\,b\,e_1$-path, and *L* is $\,b \, e_2$-path, such that *str*(*S*) is a proper subsequence of *str*(*L*).

In both cases, *S* is called the *short side* of the bubble, and *L* the *long side*.

Subsequence bubbles are useful for giving a characterization of which *st*-paths correspond to common subsequences that are not maximal, under certain hypotheses:

#### Lemma 3

Let *D* be an approximate MCS index, and let *P* be an *st*-path of *D*. Then, *str*(*P*) is a non-maximal common subsequence if and only if there exists a closed subsequence bubble *B* such that *P* traverses the *short* side *S* of *B*.

#### Proof

If an *st*-path *P* traverses *S*, then *str*(*P*) cannot be maximal: let the endpoints of *B* be *b*, *e*; the path given by the prefix of *P* up to *b*, then *L*, and then the suffix of *P* from *e* to *t* defines a common subsequence which has *str*(*P*) as a proper subsequence.

Vice versa, let *str*(*P*) be a common subsequence that is not maximal. Then, there exists an *st*-path *Q* such that *str*(*P*) is a proper subsequence of *str*(*Q*), since every *MCS* is represented in the index. Let $b\in V$ be the first node after which *str*(*P*) and *str*(*Q*) differ; it is well-defined since the first node of both paths is *s*. Symmetrically, it is well-defined the first node after *b* which belongs to both paths, which we call *e*, since both paths end at the same node *t*. Then, the subpaths $P'$ of *P* and $Q'$ of *Q* between nodes *b* and *e* form a subsequence bubble, with *P* traversing the short side: $P'\ne Q'$ since they differ at the node after *b*, and $str(P')$ is a proper subsequence of $str(Q')$ since they start and end at the same nodes, and thus positions in the strings, with at least one more symbol appearing in $Q'$. $\square$

As mentioned before, *G* is an approximate MCS index and thus satisfies the hypotheses of Lemma 3. To conclude the proof of correctness of the construction procedure $\textsc {McConstruct}$, it is sufficient to show that whenever we have an open bubble, we never add a node and edges which “close it”.

The first step towards this is the *subsequence mapping* $\lambda$ between the short side and the long side of a subsequence bubble, to couple nodes that correspond to the same characters in the subsequences. Given a subsequence bubble (open or closed), let $S=b \rightarrow v_1 \rightarrow v_2 \rightarrow \dots \rightarrow v_h$ be its short side and $L=b \rightarrow w_1 \rightarrow \dots \rightarrow w_k$ be its long side. We will use the order $v_i < v_{i+1}$ and $w_j < w_{j+1}$, which is well-defined by the corresponding relationship between the associated matches, by (4) of Theorem 1. We thereby define the injective mapping $\lambda : S \rightarrow L$ such that $\lambda (v_i) = \min \{w\in L \ \mid \ \ell (w) = \ell (v_i) \text { and } \lambda (v_{i-1}) < w\},$ where $\lambda (v_0)$ is improperly considered equal to *b*. In other words, it is a correspondence between the characters of the short string and the ones of the longer string, indicating the subsequence relationship.

We say that two matches $m^1,m^2$ are *crossing* if neither $m^1 \le m^2$ nor $m^2 \le m^1$. The subsequence mapping has the following properties:

#### Lemma 4

Let *v* be a node along the short side *S* of a subsequence bubble of the output graph of $\textsc {McConstruct}$ *G*. Then, if $v \ne \lambda (v)$, $\forall m^s \in m(F(v))$ and $\forall m^l \in m(F(\lambda (v)))$, it holds that $m^l \ne m^s$, and that either $m^s \le m^l$ or $m^s$ and $m^l$ are crossing. In particular, note that the first condition is equivalent to $F(v) \cap F(\lambda (v)) = \emptyset$.

#### Proof

Let the short side be $S=b,v_1,\dots ,v_h$, and the long side $L= b,w_1,\dots ,w_g$. We split the proof according to whether *v* is the first node of the bubble, or not.

First, consider $$v=v_1$$. By definition, *v* and $$\lambda (v)$$ are labeled with the same symbol. By determinism of *G*, $$\lambda (v)$$ $$\ne w_1$$. Therefore, for each $$z\in F(\lambda (v))$$ there are nodes $$q\in F(b), y\in F(w_1)$$ such that $$m(q)< m(y) < m(z) =: m^l$$. By construction *z* is filtered, that is $$z \not \in F(u)$$, for all nodes *u* out-neighbors of *b*, and in particular $$z \not \in F(v)$$. Thus, $$m^l$$ is different from any match in *F*(*v*). Let now $$x\in F(v)$$, and $$m^s = m(x)$$. If we had $$m^l \le m^s$$, then we would have $$m(q)< m(y) < m^s$$, which again is impossible by construction. Therefore, either $$m^s$$ crosses $$ m^l$$, or $$m^s \le m^l$$.

Let us now prove the inductive case. Assume that the thesis holds for all $1 \le j< i$, and let $v = v_i$. Suppose by contradiction that there exist $x \in F(v_i)$ and $z \in F(\lambda (v_{i}))$ such that $m^l:=m(z) \le m(x) =: m^s$. Since there are paths from $v_{i-1}$ to $v_{i}$, and from $\lambda (v_{i-1})$ to $\lambda (v_{i})$, we have that there exist $x'\in F(v_{i-1}),z' \in F(\lambda (v_{i-1}))$ such that $m(x') < m(x) = m^s$ and $m(z') < m(z) = m^l$. By contradiction hypothesis, $m(z') < m^l \le m^s$, and thus we have $m(x'),m(z') < m^s$. This contradicts the rightmost property of *A*: indeed, consider the match given by the maximum of the coordinates of $m(x'),m(z')$, $m'' = (\max (m(x')_i, m(z')_i))_{i=1}^{k}$. This match is different from $m(x')$ by induction hypothesis, as it does not hold that $m(z') \le m(x')$. Thus, $m(x') \le m'' < m^s$, so $x'$, which is the in-neighbor of *x* for character $\ell _A(x')$, is not associated with the rightmost match, a contradiction. $\square$

#### Theorem 2

The graph $G=(V,E,\ell )$ obtained by $\textsc {McConstruct}$ does not contain any closed bubbles.

#### Proof

By contradiction, let $S=b,v_1,\dots ,v_h,e$ and $L=b,w_1,\dots ,w_g,e$ be respectively the short and long sides of a subsequence bubble in *G*. We show that the edge $(v_h,e)$ could not have been added to *G* during construction. By contradiction, assume that it happens. Let $\lambda (v_h) = w_i$ and let $y\in F(e)$ and $m^e:=m(y)$ the match for *y*. Since we have edge $(v_h,e)$ in *G*, by construction we have $x \in F(v_h)$ such that $(x,y) \in E_A$. We have no occurrences of $\ell _A(x)$ between *m*(*x*) and $m^e$. Furthermore, we have $m(v_h) \le m(x)$ by definition. Now, consider node $\lambda (v_h) = w_i$, which has the same associated label $\ell _A(x)$. Since there is a path from $w_i$ to *e*, we can choose node $x'\in F(w_i)$ such that we have a path from $x'$ to *y* in *A*. In particular, this implies that $m(x') < m(y) = m^e$. By Lemma 4, we have $x'\ne x$, and we further have either $m(x') \ge m(x)$, or $m(x')$ crossing *m*(*x*). Both lead to a contradiction: for some $j \in [1..k]$, there would be an occurrence of character $\ell _A(x) = \ell _A(x')$ in $\mathcal {Z}_j[m(x)_j+1]\dots \mathcal {Z}_j[m^e_j-1]$. $\square$

**Algorithm 3:**
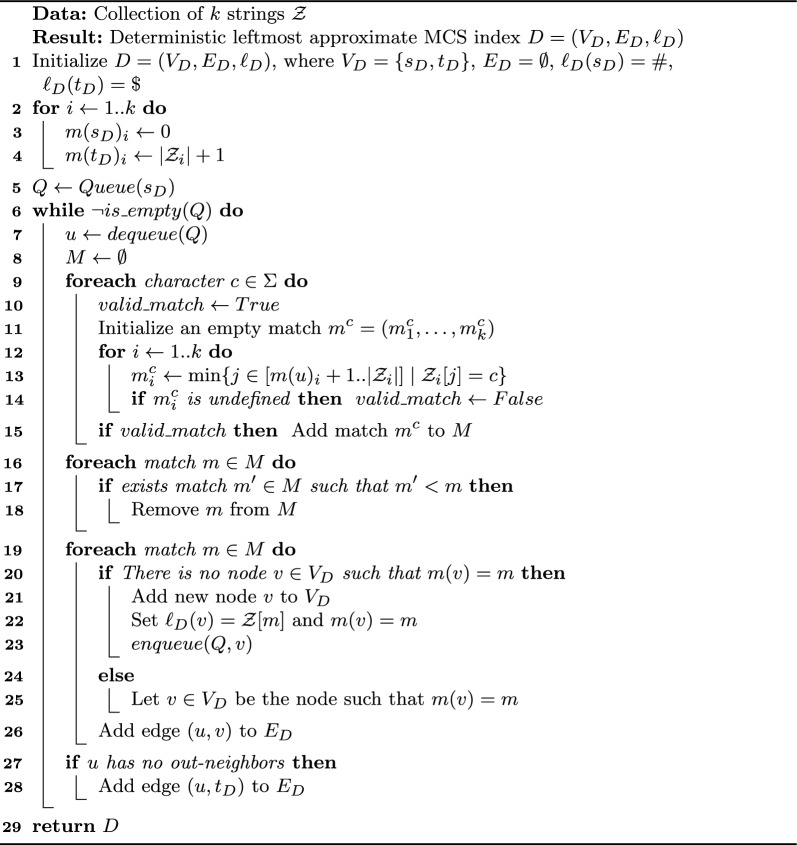
$$\textsc {CSA-mixed}$$ construction

## The McDag index

In this section, we describe how to create a smaller and improved version of $\textsc {CSA-all}$, called $\textsc {CSA-filtered}$, which filters out some non-maximal subsequences while retaining all maximal ones. As we observed in Sect. 2.2, using the smaller approximate index $\textsc {CSA-filtered}$ as input of the $\textsc {McConstruct}$ algorithm could potentially save significant computation time. This refined index is built in two steps, going through a further intermediate index $\textsc {CSA-mixed}$, as described in Sect. 3.1. Our final index $\textsc {McDag}$ is defined as the output of $\textsc {McConstruct}$ (Algorithm 2) with input $\textsc {CSA-filtered}$. Its correctness immediately follows from the correctness of $\textsc {CSA-filtered}$ (Sect. 3.2) and of $\textsc {McConstruct}$ (Sect. 2.3).

### CSA-filtered

The rationale behind $\textsc {CSA-filtered}$ is that every approximate index must contain all MCSs; thus, if several are available, we can identify portions that are not present in one reference approximate MCS index, and skip them in the construction of $\textsc {CSA-filtered}$. As a further optimization, we can also identify and avoid edges that are not traversed by any path corresponding to an MCS. To build $\textsc {CSA-filtered}$ we act in two phases: we first build a deterministic leftmost approximate MCS index called $\textsc {CSA-mixed}$, which we then use to filter out many of the contained non-maximal common subsequences in $\textsc {CSA-all}$. Both in the deterministic and in the co-deterministic version of the approximate index, we retain a property for which all nodes have distinct matches: if $m(u) = m(v)$ then $u = v$.

To build $\textsc {CSA-mixed}$, we apply a slight modification of Algorithm 1 on input strings $\mathcal {Z}$, and we obtain a labeled graph $D=(V_D,E_D,\ell _D)$: strings $\mathcal {Z}_i$ are read left-to-right, and *D* is constructed starting from its source $s_D$. Each node, except for the sink $t_D$, is visited once and its out-neighbors are added during the visit. As in the construction of $\textsc {CSA-all}$, when visiting node *u*, for each character $c \in \Sigma$ we find the closest match $m^c$ such that $m(u) < m^c$, $\mathcal {Z}[m^c] = c$, and no occurrence of *c* appears in the substring $\mathcal {Z}_i[m(u)_i+1]..\mathcal {Z}_i[m^c_i-1]$, for all *i*. At this point, if there exists another match $m'$ such that $m(u)< m' < m^c$, we discard $m^c$. Otherwise, we create *v* with $m(v) = m^c$ and $\ell _D(v) = c$ (unless such a *v* already existed), and we add the edge (*u*, *v*). If no outgoing edge was added to *u* during its visit, we add edge $(u,t_D)$.

The pseudocode can be found in Algorithm 3. The idea behind the filter is that, if $m(u)< m' <m^c$, we are guaranteed that any path that jumps directly from *u* to a node associated with $m^c$ cannot be maximal, as witnessed by match $m'$, so we do not add such edge. By disregarding $m^c$ we are thus reducing the number of unnecessary edges in the index, as well as possibly a few nodes.

**Complexity. ** We have that $|V_D| \le n^k + 2$ and $|E_D| \le |\Sigma |(n^k + 1)$ as the maximum in-degree is $|\Sigma |$. Due to the filtering operation over matches, the construction time is $O(n^k |\Sigma |^2 k)$, which is slightly higher than the one of $\textsc {CSA-all}$. The space complexity is $O(n^k |\Sigma |)$.

We now discuss the second phase, shown in Algorithm 4, which takes as input DAG *D* that corresponds to $\textsc {CSA-mixed}$ and outputs a co-deterministic rightmost approximate MCS index $A = (V_A,E_A,\ell _A)$, corresponding to $\textsc {CSA-filtered}$. As in the previous case, we begin by inserting the source $s_A$ and the sink $t_A$. In this case, we read the strings $\mathcal {Z}_i$ right-to-left, we build the graph from the sink backward, and we associate to each node *u* of *A* a set of nodes of *D*, namely *F*(*u*). Intuitively, the procedure constructs *A* from scratch by using *D* as a guide, using the following observation: for any node *x* to be in *F*(*u*) there must exist an $x\,t_D$-path and a $u\,t_A$-path that spell out the same strings. This means that a node *u* is associated with a set of rightmost suffixes of common subsequences, given by the equivalent paths. Equivalently, each in-neighbor of the nodes in *F*(*u*) is associated with a set of rightmost prefixes, that can concatenate to any suffix of *u* to create a string of the language of *D*. So, if we find a match *m* between any such in-neighbor *x* and *u* (i.e. $m(x)< m < m(u)$), then all concatenations of the prefixes of *x* and the suffixes of *u* are not maximal. Hence, we can discard *x* from the set of nodes that are modeled by the in-neighbors of *u*. This can potentially remove all the nodes of a given label from the in-neighbors of *F*(*u*), thus reducing the number of edges that we add to *A*.

We set $F(s_A) = \{s_D\}$, $F(t_A) = \{t_D\}$, $m(s_A) = m(s_D)$ and $m(t_A) = m(t_D)$. For the construction, we introduce relation $\prec$ between matches, which is used to define a priority queue.

#### Definition 6

Let *m* and $m'$ be two matches. We say $m \prec m'$ if and only if $\max _i m_i < \max _j m'_j$.

We use the priority queue in Algorithm 4 so to ensure that each node *u* is visited only after all other nodes *v* with associated matches $m(u) \prec m(v)$ have already been visited. In other words, we visit the nodes in an inverse topological order, even when not all nodes have been created. This property is important because all out-neighbors of *u* contribute to the definition of *F*(*u*), and we want *F*(*u*) to be completely defined before visiting *u*. Since we are building an approximate MCS index, property 4 must hold, so for all nodes *w* that have matches $m(w) \prec m(u)$, we have that $m(u) \not < m(w)$, so *w* cannot be an out-neighbor of *u*.

We visit all nodes exactly once, except for $s_A$, starting from $t_A$. During the visit to the generic node *u*, we define a set containing the labels of some of the in-neighbors of the nodes of *D* that are in set *F*(*u*). Specifically, we choose nodes *x* such that there is a $y \in F(u)$ and $(x,y) \in E_D$, and for which there exists no match *m* such that $m(x)< m < m(u)$. Finally, we build one *rightmost* match $m^c$ for each one of the selected labels *c*, and, if there is no node $v \in V_A$ with associated match $m(v) = m^c$, we create it and set $\ell _A(v) = c$; in all cases, we add (*v*, *u*) to the list of edges $E_A$. Instead, if no label is selected and *u* is not assigned any in-neighbors, we add edge $(s_A,u)$ to $E_A$.

**Complexity. ** Again, *A* has as many nodes as the matches involved in its construction, plus $s_A$ and $t_A$. We have that $|V_A| \le n^k + 2$ and $|E_A| \le |\Sigma |(n^k + 1)$ as the maximum in-degree is $|\Sigma |$.

Before proving the correctness of the construction of $\textsc {CSA-filtered}$, we describe a further optimization that we can perform when the number of strings is $k=2$.

**Algorithm 4:**
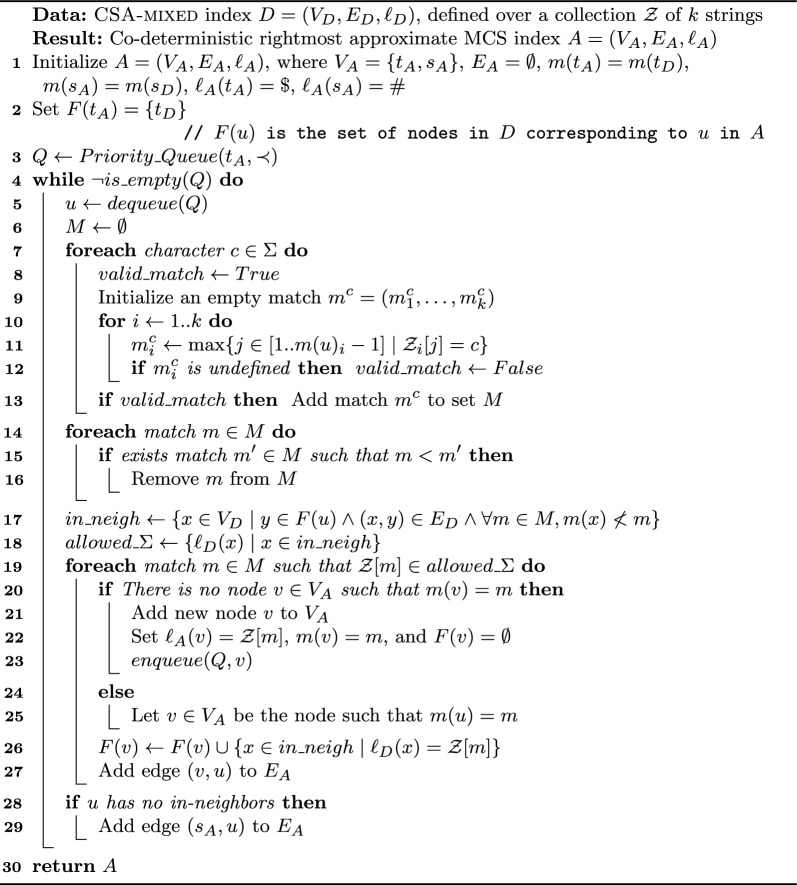
$$\textsc {CSA-filtered}$$ construction

**Optimization for **$k=2$ **strings** We can apply an optimization to lower the time complexity of Algorithm 3 and Algorithm 4. Both algorithms make comparisons between matches to filter out the *dominated* ones. Specifically, for a node *u* and a set of candidate matches *M*, when we are building a leftmost MCS approximate index (Algorithm 3), we say $m \in M$ is dominated by $m' \in M$ if $m(u)< m' < m$. Symmetrically, when building a rightmost MCS index (Algorithm 4), $m'$ dominates *m* if $m< m' < m(u)$.

For the general case of more than two strings, we apply the baseline algorithm of checking all distinct pairs of matches from *M*, as presented in the pseudocodes; this takes $O(k|M|^2)$ time. In the case of $k=2$ we can do this faster: first, we sort lexicographically the set of matches *M*: we use increasing order for the case of the leftmost MCS index and decreasing order for the rightmost case. For a given match $m'$, all matches *m* of *M* that come after $m'$ in the order cannot dominate $m'$, as the first coordinate of *m* surely is not *between* the first the coordinate of $m'$ and the first one of *m*(*u*). Using this property, in the leftmost case we can record the minimum value of the second coordinate found so far: if a later match *m* has the second coordinate that is greater than this minimum, then the match $m'$ that defined such minimum value dominates *m*, i.e. $m(u)<m'<m$. Symmetrically, for the rightmost case, we can record the maximum value of the second coordinate, and discard matches that have the second coordinate lower than the maximum.

**Complexity. ** Depending on the sorting algorithm used, this procedure may take $O(|M|+n)$ or $O(|M|\log |M|)$ time, which guarantees an improvement over the baseline $O(|M|^2)$.

### Correctness of CSA-filtered

#### Lemma 5

The resulting $\textsc {CSA-mixed}$ *D*, output of Algorithm 3, is a deterministic leftmost approximate MCS index.

#### Proof

By construction, each edge (*u*, *v*) is in graph *D* only if $m(u) < m(v)$, so *D* is acyclic by the antisymmetry of <. This also implies that all strings encoded in an *st*-path $s_D, u_1,\dots ,u_h,t_D$ are common subsequences of $\mathcal {Z}$, as there exists a matching $m(u_1),\dots ,m(u_h)$. Properties 1 and 2 are trivially granted by construction: in particular, all nodes except for $t_D$ are visited, so they are assigned some out-neighbors (they are not source), and whenever a node different from $s_D$ and $t_D$ is added, it is assigned an in-neighbor (that node is not a sink). Since *D* is acyclic and not empty, at some point there must exist a node with no out-neighbors, which is immediately assigned as an in-neighbor of $t_D$. This lets us conclude that $s_D$ and $t_D$ are the only source and sink. By construction if there is an edge (*u*, *v*) in *D*, then for all $i \in [1..k]$, $m(v)_i$ is chosen as the first occurrence of $\ell _D(v)$ in $\mathcal {Z}_i$ after $m(u)_i$, so *D* is leftmost. Also, each node is visited once and at most one out-neighbor per character is added, so *D* is deterministic.

Finally, we must show that all MCSs are encoded in *D*. Suppose by contradiction there is a string $W \in MCS(\mathcal {Z})$ for which there is no path in *D* that spells it. Let $P = s_D,u_1,\dots ,u_j$ the path that spells the longest prefix of *W*; by determinism *P* is unique. Let $m'$ be the leftmost match for character $W[j+1]$ to the right of $m(u_j)$. $m'$ must exist because *W* is a common subsequence and, since *D* is leftmost, for each *i*, $m(u_j)_i$ defines the shortest prefix of $\mathcal {Z}_i$ that contains $W[1]\dots W[j]$. Since there is no out-neighbor of $u_j$ with associated match $m'$, there must exist a match *m* such that $m(u_j)< m < m'$. Consider the rightmost matching $(m^i)_{i=1}^{|W|}$ of *W*, i.e. the one where each $m^{i}$ is chosen to contain the rightmost occurrences of character *W*[*i*] before match $m^{i+1}$. For *W* to be a common subsequence it must hold that $m' \le m^{j+1}$. But if this is true, then we can build string $\mathcal {Z}[m(u_1)]\dots \mathcal {Z}[m(u_j)]\mathcal {Z}[m]\mathcal {Z}[m^{j+1}]\dots \mathcal {Z}[m^{|W|}]$, a supersequence of *W*. This contradicts the maximality of *W*, so we are done. $\square$

#### Lemma 6

The resulting $\textsc {CSA-filtered}$ *A*, output of Algorithm 4, is a co-deterministic rightmost approximate MCS index.

#### Proof

By construction, for every edge (*u*, *v*) in *A*, we have that $m(u) <m(v)$, so *A* must be acyclic by the antisymmetry of the < operator. Again by construction, properties 1 and 2 of approximate MCS indices must hold: every time a node $u \not \in \{s_A,t_A\}$ is added to *A*, it is associated with match *m*(*u*) and label $\ell _A(u) = \mathcal {Z}[m(u)]$. Moreover, since an edge (*u*, *v*) is immediately added to $E_A$, for some other node *v*, it means that *u* is not a sink. Nodes $s_A$ and $t_A$ are added at the beginning, and have matches $m(s_A)_i = 0$ and $m(t_A)_i = |\mathcal {Z}_i|+1$ for all $i \in [1..k]$, and $\ell _A(s_A) = \#$ and $\ell _A(t_A) = \$$. Incoming edges are only added to a node when it is extracted from the queue and visited. As node $s_A$ is the only node that is never explicitly visited ($\ell _A(s_A) \not \in \Sigma$), it does not have any incoming edge and thus it is the unique source. Moreover, $\ell _A(t_A) \not \in \Sigma$, so node *t* cannot be added as an in-neighbor of any other node. Since *A* is acyclic and all other nodes except $s_A$ are visited once, at least one of them must not have any in-neighbors, so it is added as an out-neighbor of $s_A$, meaning $t_A$ is the only sink.

Each node has at most one in-neighbor per character, so *A* is co-deterministic, and no two distinct *st*-paths can spell out the same string. Consider path $P=s_A,u_1,\dots ,u_h,t_A$ in *A*, and let $str(P) = \ell _A(u_1),\dots ,\ell _A(u_h)$ be the unique string associated with *P*. By construction we have that if *A* contains edge (*u*, *v*), with $u \ne s_A$ and $v \ne t_A$, then $m(u)_i < m(v)_i$ for each $i \in [1..k]$, so $m(u_1)< \dots < m(u_h)$. This is enough to prove that *str*(*P*) is a common subsequence of $\mathcal {Z}$ with associated matching $(m^1,\dots ,m^h)$. Moreover, we have that $m(u)_i$ is chosen to be the maximum position smaller than $m(v)_i$ such that $\mathcal {Z}_i[m(u)_i] = \ell _A(u)$, hence *A* is rightmost.

Suppose by contradiction there is a string $W \in MCS(\mathcal {Z})$ that is not spelled out by any *st*-path. Let $v_{j} \in V_A$ be the node that corresponds to the longest suffix of *W* present in *A*. By co-determinism, we have a unique path $P=v_{j},\dots ,v_{|W|},t$ such that $str(P) = W[j]\dots W[|W|]$, and there is no node *u* with label $\ell _A(u) = W[j-1]$ such that $(u,v_{j}) \in E_A$.

Consider the leftmost matching $(m^i)_{i=1}^{|W|}$ of *W*, i.e. the one where each $m^{i+1}$ is composed of the leftmost occurrences of character $W[i+1]$ after match $m^i$; also, consider the rightmost match $m'$ of character $W[j-1]$ before $m(v_{j})$. For *W* to be a common subsequence it must hold that $m^{j-1}\le m'$. By maximality of *W* there cannot be any match $m''$ such that $m'< m'' < m(v_{j})$, or there would exist another common subsequence $W' = \mathcal {Z}[m^1]\dots \mathcal {Z}[m^{j-1}]\mathcal {Z}[m'']str(P)$, a supersequence of *W*. This means that during construction, match $m'$ has not been discarded from set *M*. The only way not to add edge $(u,v_j)$, with $m(u) = m'$, is for $\mathcal {Z}[m']$ not to be in the set $allowed\_\Sigma$.

By construction, *F*(*v*) is the set of nodes of *D* that have the same label of *v* and are in-neighbors of a node in *F*(*w*), for some $(v,w) \in E_A$. By setting $F(t_A) = \{t_D\}$ as a base case, we have that *F*(*v*) is the set of nodes of *D* from which starts a path that spells out the same string as one of the paths that start from *v* in *A*. To not have $\mathcal {Z}[m']$ in the set $allowed\_\Sigma$, it means that for all nodes $y \in F(v_j)$ there is no node *x* such that $(x,y) \in E_D$, $\ell _D(x) = \mathcal {Z}[m']$ and for all matches *m* in *M*, $m(x) \not < m$. But since *D* is a deterministic approximate MCS index, there must be a unique path $s_D,x_1,\dots ,x_{|W|},t_D$ that spells out *W*, so $x_j \in F(v_j)$, $\ell _D(x_{j-1})=\mathcal {Z}[m']$ and there must exist a match $m'' \in M$ such that $m(x_{j-1}) < m''$. But this contradicts the maximality of *W*, as we can build $\ell _A(x_1)\dots \ell _A(x_{j-1})\mathcal {Z}[m'']str(P)$, a supersequence of *W*. $\square$

## Experimental analysis

In this section, we report experiments conducted to empirically assess the complexity of the proposed algorithms. We begin by describing the experimental setup and datasets used. The analysis is organized into two parts: the first part focuses on the case of $k=2$ strings, which is of particular interest in the literature [8, 15]. Here, we provide a comprehensive analysis of index sizes and construction times. The second part addresses the generalization to $k \ge 2$ strings, investigating how the index size scales for various values of *k* for both the minimum deterministic MCS index ($\textsc {MCS-minimized}$) and $\textsc {McDag}$, relative to the $n^k$ curve.

### Experimental setup

Our algorithms are implemented in C++ using g++ 11.4.0 and compiled with the -O3 and –march=native flags. All tests were conducted on a DELL PowerEdge R750 machine in a non-exclusive mode. This platform features 24 cores with 2 Intel(R) Xeon(R) Gold 5318Y CPUs at 2.10 GHz and 989 GB of RAM. The operating system is Ubuntu 22.04.2 LTS.

**Datasets.** To evaluate the effectiveness of our methods, we selected two datasets:

- *random *: Random sequences of variable alphabet size generated using the uniform distribution from the standard C++ library. The generated strings in our experiments have length 3000.
- *hiv-1 *: 43 complete HIV-1 genomes, referenced in the literature [28]. The average length of such sequences is 9267 base pairs.

Other real-world and synthetic datasets were used in preliminary experiments, but the results resembled the ones we discuss in this section; for the sake of simplicity, we report results mainly from the $\mathtt {hiv{-}1}$ dataset.

**Index data structures.** We implemented the following indexing data structures in C++ and evaluated them based on the number of nodes, edges, and construction time:

- $\textsc {M-Dag}$: The DAG storing MCSs presented in [8].
- $\textsc {CSA-all}$: The Common Subsequence Automaton [5] storing all common sequences, both non-maximal and maximal, and implemented as a labeled DAG (built by Algorithm 1).
- $\textsc {CSA-maximal}$: The MCS index built upon $\textsc {CSA-all}$ using $\textsc {McConstruct}$.
- $\textsc {CSA-filtered}$: An optimized version of $\textsc {CSA-all}$, which filters out many non-maximal common subsequences during construction, as presented in Sect. 3.1 (Algorithm 4).
- $\textsc {McDag}$: Our proposed MCS index, built upon $\textsc {CSA-filtered}$ using $\textsc {McConstruct}$.
- $\textsc {MCS-minimized}$: The minimized version of $\textsc {M-Dag}$, $\textsc {CSA-maximal}$, and $\textsc {McDag}$ that we can compute starting from any such MCS index using Revuz’s algorithm [29]. Note that, as these three DAGs encode the same language, when minimized they converge to the unique optimal index $\textsc {MCS-minimized}$.

In the experiments that follow, we focus on evaluating the size of index $\textsc {McDag}$, which, by definition of minimal automaton, is always larger than $\textsc {MCS-minimized}$. For practical applications, $\textsc {MCS-minimized}$ is clearly a better index, but the time and space needed to compute it strongly depend on which MCS index we start from.^2^ In Sect. 4.2.2 we will take a closer look at the computational paths that allow us to reach the minimal index.

### Results on $k=2$ strings

#### Index size

**Fig. 5:**
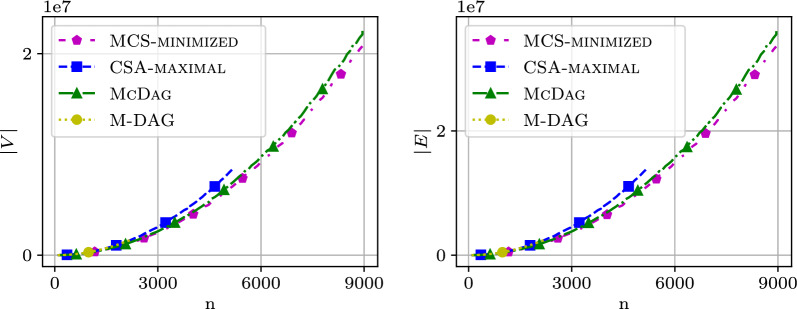
Number of nodes and edges in the DAGs on the dataset $\mathtt {hiv{-}1}$

We analyzed the computational cost of the index data structures $\textsc {M-Dag}$, $\textsc {CSA-maximal}$, $\textsc {McDag}$, and $\textsc {MCS-minimized}$ in terms of the number of nodes, edges, and construction time. Figure 5 shows two plots: the left plot displays the number of nodes and the right plot shows the number of edges for $\textsc {M-Dag}$, $\textsc {CSA-maximal}$, $\textsc {McDag}$, and $\textsc {MCS-minimized}$ as the sequence length *n* increases. The x-axis reports the sequence length *n*, while the y-axis reports the corresponding number of nodes/edges. For the following figures, we used two sequences from the $\mathtt {hiv{-}1}$ dataset, namely AF005496 and K03454.

$\textsc {M-Dag}$ consistently has more nodes/edges than $\textsc {CSA-maximal}$, which in turn has more than $\textsc {McDag}$. The computation for the former two was interrupted after a timeout of 8000 s (giving a truncated plot, respectively, for $n = 2300$ and $n = 5700$), while $\textsc {McDag}$ completed in less than 600 s. This performance gap, when comparing $\textsc {CSA-maximal}$ and $\textsc {McDag}$, is due to the smaller size of $\textsc {CSA-filtered}$ (the input approximate index for constructing $\textsc {McDag}$) with respect to $\textsc {CSA-all}$.

**Fig. 6:**
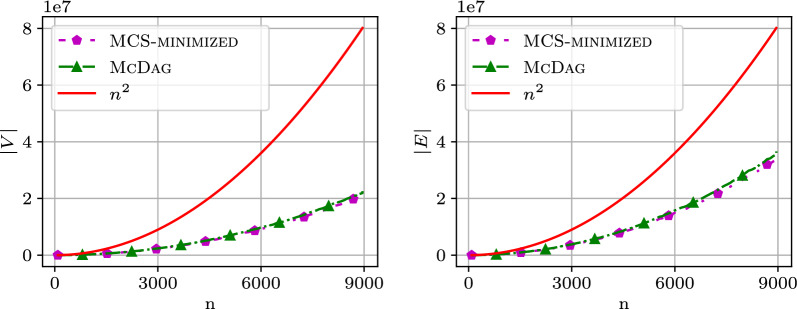
Number of nodes and edges for $\textsc {McDag}$ and $\textsc {MCS-minimized}$, in comparison with $n^2$, computed on the $\mathtt {hiv{-}1}$ dataset

All plotted curves are below $n^2$, empirically established for all of our datasets. We plot the data for $\textsc {McDag}$ and $\textsc {MCS-minimized}$, along with the curve for $n^2$ to ease the comparison in Fig. 6. $\textsc {McDag}$ is the closest to $\textsc {MCS-minimized}$, with nodes/edges only 4-7% more than $\textsc {MCS-minimized}$ (compared to $\textsc {M-Dag}$’s 26-31% and $\textsc {CSA-maximal}$’s 19-27%).

It remains an open problem to prove that the number of nodes and edges in $\textsc {McDag}$ is always $< n^2/c$ for some constant $c > 0$, regardless of the sequence alphabet. Synthetic sequences $\mathcal {Z}_1$ and $\mathcal {Z}_2$ of length *n* can be defined to yield $\Omega (n^2)$ nodes and edges in $\textsc {McDag}$, but we found no real-world or synthetic sequences exceeding $n^2$ nodes or edges for $k=2$.

**Fig. 7:**
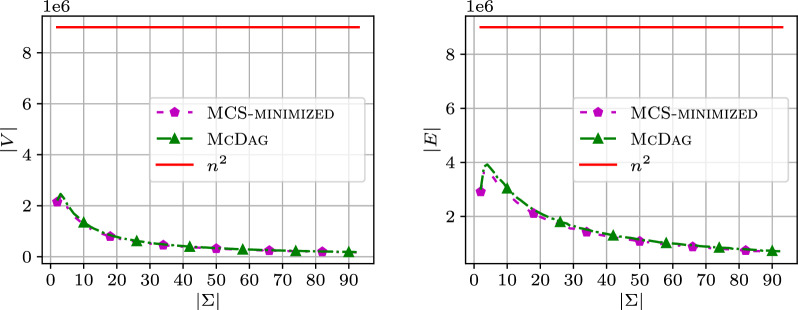
Number of nodes and edges in $\texttt{random}$ for increasing alphabet size $|\Sigma |$, vs $n^2 = 9\cdot 10^6$

To consider different alphabet sizes, in Fig. 7, we fix $n=3000$ on $\texttt{random}$ data, and report the number of nodes and edges for $\textsc {McDag}$ and $\textsc {MCS-minimized}$ for varying $|\Sigma |$ compared to $n^2 = 9 \cdot 10^6$.

#### Construction time

**Fig. 8:**
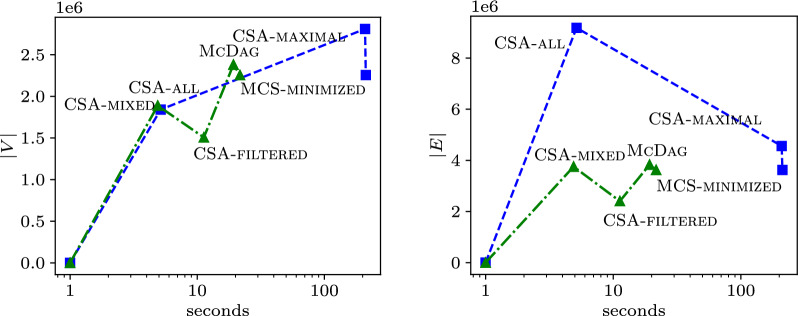
Filtering out and construction time on the $\mathtt {hiv{-}1}$ dataset

We analyze the time performance of the computation paths that allow us to reach different MCS indices. In the plots of Fig. 8, we compare two approaches:

- The blue path is the one in Sect. 2: we start from $\textsc {CSA-all}$, obtain $\textsc {CSA-maximal}$, and then, using Revuz’s algorithm, minimize to obtain $\textsc {MCS-minimized}$.
- The green path is its optimized version: we start from approximate index $\textsc {CSA-mixed}$ (which is symmetrical to $\textsc {CSA-all}$ and takes the same time), obtain $\textsc {CSA-filtered}$, get $\textsc {McDag}$, and then $\textsc {MCS-minimized}$ using Revuz’s algorithm, as in the blue path.

The x-axis reports, in logarithmic scale, the time expressed in seconds, and the y-axis reports the number of nodes in the left plot, and the number of edges in the right plot, both in linear scale. The plotted data is obtained from the two aforementioned genomes of dataset $\mathtt {hiv{-}1}$ truncated to 3000 characters. We can see how the blue path to obtain $\textsc {McDag}$ scales better in both time and space and can be used to build $\textsc {MCS-minimized}$ more efficiently.

**Fig. 9:**
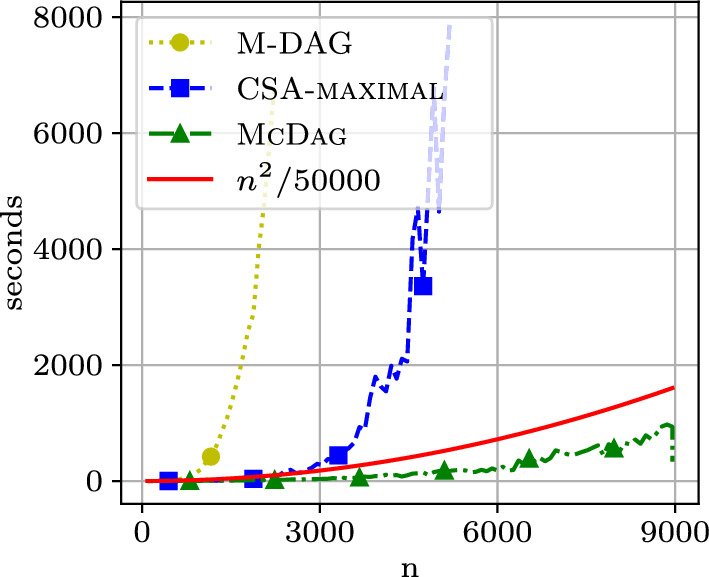
Construction time on the $\mathtt {hiv{-}1}$ dataset

Fig. 9 shows the running times for constructing the DAGs. All methods were implemented as fairly as possible. $\textsc {McDag}$ scales well compared to the plot of $n^2/50000$, which is significant given that $\textsc {McDag}$ also computes $LCS(\mathcal {Z}_1,\mathcal {Z}_2)$, which has a quadratic conditional lower bound for computation [3, 4].

On the other hand, the previous figures show that the optimizations employed to build $\textsc {CSA-filtered}$ deliver significant improvements to the construction time: this finds justifications in the lower amount of edges that $\textsc {CSA-filtered}$ has with respect to $\textsc {CSA-all}$, as well as the actual size of the final data structure, after $\textsc {McConstruct}$. Indeed, we repeated the experiment for 100 random pairs of elements in the $\mathtt {hiv{-}1}$ dataset (truncated to length 3000). We report the mean values obtained (± standard deviation): over all such experiments, we obtained an average of 1.83 million nodes (± 17k) and 9.12 million edges (± 86k) for $\textsc {CSA-all}$, and of 1.48 million nodes (± 17k) and 2.37 million edges (± 25k) for $\textsc {CSA-filtered}$. This showcases the aforementioned expected significant decrease in the number of edges (as well as a decrease in the number of nodes) as a result of the filtering procedure. For the two final indices, we obtain an apparently less drastic difference: the number of nodes and edges of $\textsc {CSA-maximal}$ are respectively 2.77 million (± 32k) and 4.52 million (± 55k) on average, while for $\textsc {McDag}$ we obtain an average of 2.34 million nodes (± 29k) and 3.79 million edges (± 48k). The real difference here is instead reflected by the total size of the grouped nodes, given by the quantity $\sum _{v\in V} |F(v)|$: indeed, as we observed in Sect. 2.2, the cumulative size of the sets *F*(*v*) is the main contributor of the complexity of the $\textsc {McConstruct}$ procedure. For this figure, we obtain a cumulative size of 18.21 million (± 355k) for $\textsc {CSA-maximal}$, and of 7.21 million (± 125k) for $\textsc {McDag}$, explaining the substantial difference in the runtime of procedure $\textsc {McConstruct}$ with respect to the different input indices.

**Fig. 10:**
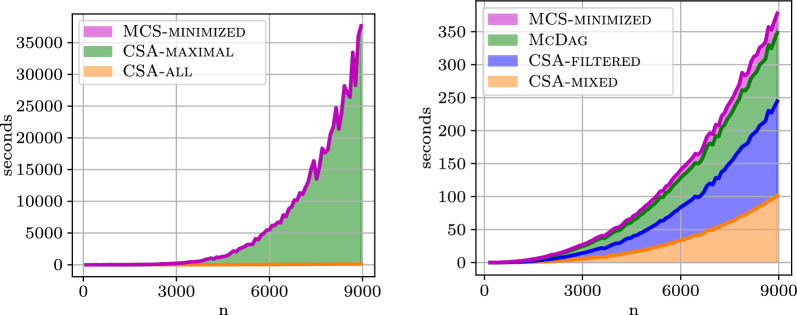
Stacked plots of the running time for the computational paths of $\textsc {CSA-maximal}$ (left) and $\textsc {McDag}$ (right) over $k=2$ strings from the $\mathtt {hiv{-}1}$ dataset

Finally, Fig. 10 illustrates the time required to complete each step in the computational paths of $\textsc {CSA-maximal}$ and $\textsc {McDag}$.

Remarkably, in the left plot, the $\textsc {McConstruct}$ procedure consumes the vast majority of computation time, far surpassing the time needed to construct $\textsc {CSA-all}$. In contrast, in the right plot, each step of the computation is balanced, with no significant bottlenecks, and the total computation times (on the y-axis) are orders of magnitude smaller than those needed for $\textsc {CSA-maximal}$. This demonstrates that the optimizations introduced in Sect. 3.1 to build a smaller initial approximate MCS index have highly effective practical consequences.

### Index size for $k\ge 2$

In this section, we examine how the size of $\textsc {McDag}$ scales with multiple input strings. Since finding the longest path in $\textsc {McDag}$ allows us to quickly compute the LCS across multiple strings—a problem known to be NP-hard [2]—we can only construct indices over shorter input strings for fixed $k>2$. To illustrate the behavior of $\textsc {McDag}$ and $\textsc {MCS-minimized}$ with multiple input strings, we present several plots for different values of *k*, using input strings from the $\mathtt {hiv{-}1}$ dataset. Specifically, we used the first *k* strings of this list: AF004885, AF005494, AF005496, AF061641, AF061642, AF067155.

**Fig. 11:**
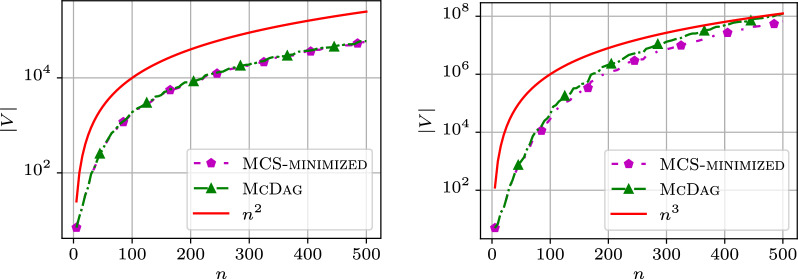
Number of nodes in $\mathtt {hiv{-}1}$ for $k=2$ sequences and $k=3$ sequences

Figure 11 shows the index sizes for $k=2$ and $k=3$. The y-axis displays the number of nodes in logarithmic scale, while the x-axis shows the length of the input strings in linear scale. As expected, the left plot shows that for two strings, both $\textsc {McDag}$ and $\textsc {MCS-minimized}$ produce a curve that follows the $n^2$ trend, confirming our observations from previous sections.

Surprisingly, when we increase the value of *k*, the behavior changes. For $k=3$, we observe a distinct growth trend on the right of Fig. 11: the shapes of the MCS index curves appear *flatter* than the theoretical $n^3$ curve. Notably, for values of *n* near 500, the $\textsc {McDag}$ curve almost meets the $n^3$ curve, and the $\textsc {MCS-minimized}$ curve follows closely.

**Fig. 12:**
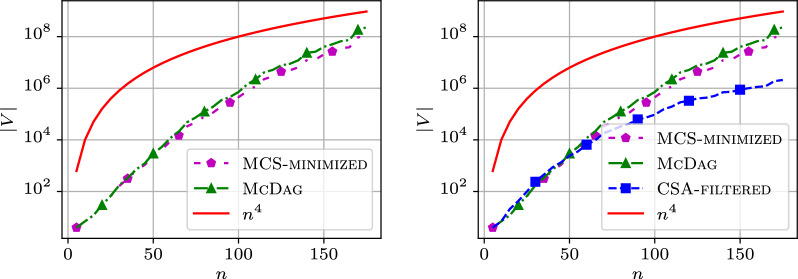
Number of nodes in $\mathtt {hiv{-}1}$ for $k=4$ sequences, compared with the size of $\textsc {CSA-filtered}$ in the plot on the right

This *flattening* effect in the curves becomes even more pronounced for $k=4$ on the left of Fig. 12. Due to scalability limitations, we could not compute results for larger values of *n*, but it seems reasonable to assume that, as with $k=3$ and sufficiently long input strings, the number of nodes for any deterministic MCS index would likely exceed $n^k$.

To better understand the reason for this unexpected growth in the number of nodes, the plot on the right of Fig. 12 introduces (in blue) the number of nodes in $\textsc {CSA-filtered}$. As can be seen, this curve follows the $n^4$ trend (in red), consistent with the size complexity of $\textsc {CSA-filtered}$ as discussed in Sect. 3.1. Comparing this with the curves for $\textsc {McDag}$ and $\textsc {MCS-minimized}$ highlights their different behaviors: the latter curves clearly grow more rapidly than $\textsc {CSA-filtered}$.

**Fig. 13:**
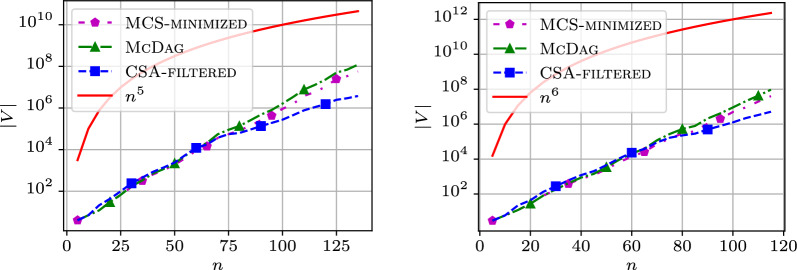
Number of nodes in $\mathtt {hiv{-}1}$ for $k=5$ sequences and $k=6$ sequences

From the plots examined so far, we observe that the reason for the *growth* in the number of nodes in $\textsc {McDag}$ and $\textsc {MCS-minimized}$ is due to the *elimination* of *non-maximal* common subsequences, which causes the creation of extra nodes and paths in the indices. This observation becomes even clearer for $k=5$ and $k=6$, as shown in Fig. 13, where the growth of the MCS indices appears linear in log scale whereas $\textsc {CSA-filtered}$ (in blue) follows the $n^k$ trend (in red).

In log scale plots like those presented in this section, a straight line represents a function that grows *exponentially* with the value on the x-axis. This suggests that for a fixed, sufficiently large *k*, the observed curves approach a function where *n* appears in some form in the exponent. Interestingly, this exponential-like behavior affects both $\textsc {McDag}$ and $\textsc {MCS-minimized}$, suggesting that *any* deterministic MCS index would grow exponentially with *n* when $k > 2$ is fixed, even using alternative algorithms to our $\textsc {McConstruct}$.

## Conclusions

In this paper, we presented a novel method for building a compact index of all maximal common subsequences (MCS) of multiple strings, called $\textsc {McDag}$, and designed to be both understandable and straightforward to implement. We empirically evaluated $\textsc {McDag}$ on synthetic data and DNA sequences from public datasets, demonstrating its effectiveness and efficiency. For two strings, $\textsc {McDag}$ provides a practical solution for applications in bioinformatics, text processing, and other fields that require efficient sequence analysis. By focusing on a co-deterministic approach and employing filtering techniques, we have shown that $\textsc {McDag}$ is a compact, precise index that handles substantial datasets, utilizing only 4-7% more than the minimum required nodes. It remains an open problem to prove whether $\textsc {McDag}$ always contains fewer than $n^2$ nodes and edges for two strings, independently of the alphabet size $|\Sigma |$.

For the general case of multiple strings, we observed that $\textsc {McDag}$ is the first method that allows for indexing all MCSs in a set of more than two strings. However, $\textsc {McDag}$ and even the smallest deterministic MCS index appear to grow exponentially in size with three or more strings. We leave open the question of whether it is possible to construct an MCS index for the general case of *k* strings that, for fixed $k>2$, has a number of nodes that is polynomial in *n*.

Finally, we plan to expand the scalability of our approach for two strings by extending the sequence length *n* through advanced space-saving techniques, parallelism, and SIMD (Single Instruction, Multiple Data) instructions. Ultimately, our goal is to provide a robust and versatile tool for MCS indexing that balances accuracy, efficiency, and practicality, advancing sequence analysis methodologies and their applications.

## Data Availability

No datasets were generated or analysed during the current study.

## Footnotes

1 Based on our understanding of [9], the DAG introduced therein could potentially fit our definition. However, we lack sufficient details to make this assertion with certainty. Also, we illustrate the case $k=2$ as we can compare $\textsc {McDag}$ to the literature.

2 For example, we can obtain $\textsc {MCS-minimized}$ starting from a deterministic MCS index modeled by a trie, but this could take exponential space in practice.
